# Appendicitis while on alectinib for non-small cell lung cancer: a tale of two case reports

**DOI:** 10.3389/fonc.2024.1398414

**Published:** 2024-09-26

**Authors:** Paul Wheatley-Price, Boaz Wong, Hely Shah, Harman Sekhon, Sara Moore

**Affiliations:** ^1^ Department of Medical Oncology, The Ottawa Hospital Cancer Centre, Ottawa, ON, Canada; ^2^ Department of Pathology and Laboratory Medicine, Eastern Ontario Regional Laboratory Association, University of Ottawa, Ottawa, ON, Canada

**Keywords:** alectinib, appendicitis, NSCLC, case report, cancer

## Abstract

**Introduction:**

Aberrant expression of anaplastic lymphoma kinase (ALK) is found in 3%–7% of patients with non-small cell lung cancer (NSCLC). Alectinib is a tyrosine kinase inhibitor used as first-line treatment targeting ALK-positive tumors. We herein report two cases of appendicitis highlighting it as a rare, possible adverse event of treatment with alectinib.

**Case presentation:**

The first case is a 60-year-old woman with a previous history of stage 1 lobular breast cancer and early-stage lung cancer treated with segmentectomy, subsequently presenting with ALK-positive advanced NSCLC. Treatment with alectinib resulted in partial response, but she developed gastrointestinal symptoms that were assessed with computed tomography (CT) of the abdomen revealing right lower quadrant stranding without appendiceal visualization. Her symptoms continued despite an antibiotic course with re-imaging concerning for acute appendicitis, which was successfully treated with appendectomy and amoxicillin–clavulanic acid. The second case is a previously healthy 58-year-old man with advanced ALK-positive NSCLC who was started on first-line treatment with alectinib and subsequently diagnosed with asymptomatic acute appendicitis on re-staging CT abdomen. Signs on CT resolved with amoxicillin–clavulanic acid. Definitive treatment was conducted with a delayed elective appendectomy. Both patients remained on alectinib over the courses of appendicitis without interruption.

**Conclusion:**

While appendicitis has not been previously described as an adverse effect of alectinib, its incidence in two patients at our center within several months following the administration of alectinib raises its suspicion as a possible adverse effect.

## 1 Introduction

Oncogenic gene fusions involving anaplastic lymphoma kinase (ALK) are present in 3%–7% of patients with non-small cell lung cancer (NSCLC). Currently, alectinib is a standard first-line choice of agent in advanced ALK fusion-positive (ALK+) NSCLC (1). As demonstrated in the 2017 phase III ALEX clinical trial, given its increased CNS-penetrating properties, alectinib showed superior efficacy and lower toxicity compared to crizotinib, the previous standard first-line therapy in patients with untreated ALK+ at that time (2). Like other receptor tyrosine kinase inhibitors, common side effects of alectinib include constipation, fatigue, peripheral edema, and muscle soreness. Further severe adverse effects include elevated creatinine kinase, hemolysis, neutropenia, and hepatotoxicity (3). Specifically to the gastrointestinal system, the ALEX trial reported nausea, diarrhea, and vomiting as common adverse effects. Patients receiving crizotinib more frequently had these gastrointestinal adverse events over grade 3 compared to alectinib (2). Similar results were found for other ALK inhibitors, brigatinib and lorlatinib, in the ALTA1L and CROWN clinical trials, respectively (4, 5). Furthermore, a systematic review and meta-analysis comparing the side effects of different ALK inhibitors reiterate nausea, vomiting, diarrhea, and constipation as the most common gastrointestinal side effects. Alectinib and loratinib had the lowest rates of these adverse events among all ALK inhibitors (6). Appendicitis was not mentioned in this analysis.

To our knowledge, there have only been two documented cases of alectinib-associated appendicitis from a pooled analysis of alectinib studies (7). Appendicitis is not currently listed as a known adverse side effect of alectinib. In this case report, we describe two cases of patients with ALK+ NSCLC developing appendicitis while receiving alectinib.

## 2 Case description

### 2.1 Case study X

Patient X was a 60-year-old woman with no smoking history, diagnosed with stage IA right lower lobe lung adenocarcinoma treated with a segmentectomy in November 2018. Surveillance computed tomography (CT) scan in February 2020, confirmed by PET scan in March 2020, identified recurrent local disease, mediastinal adenopathy, and sclerotic bone lesions. Biopsy of mediastinal adenopathy via endobronchial ultrasound (EBUS) confirmed recurrent lung adenocarcinoma. Biomarker testing revealed an ALK fusion, programmed death-ligand 1 (PD-L1) expression greater than 50%, and no EGFR/KRAS/BRAF/ROS1 alterations. Staging magnetic resonance imaging (MRI) brain scan showed multiple asymptomatic intracranial metastases.

After palliative radiotherapy to thoracic spine metastases, systemic treatment was initiated with alectinib 600 mg twice a day (BID) orally (PO) in April 2020. In both patient cases, alectinib was selected as first-line therapy given that it had the greatest efficacy of all ALK inhibitors in literature at the time of treatment (2). Additionally, at the time of the patient treatment, alectinib was publicly funded as first-line treatment, but lorlatinib was not. Within 2 weeks of starting therapy, the patient had symptomatic improvement with only a reported side effect of grade 1 peripheral edema. Repeat staging scans in June 2020 showed resolution of intracranial metastases and improvement of extracranial metastatic disease.

**Figure 1:**
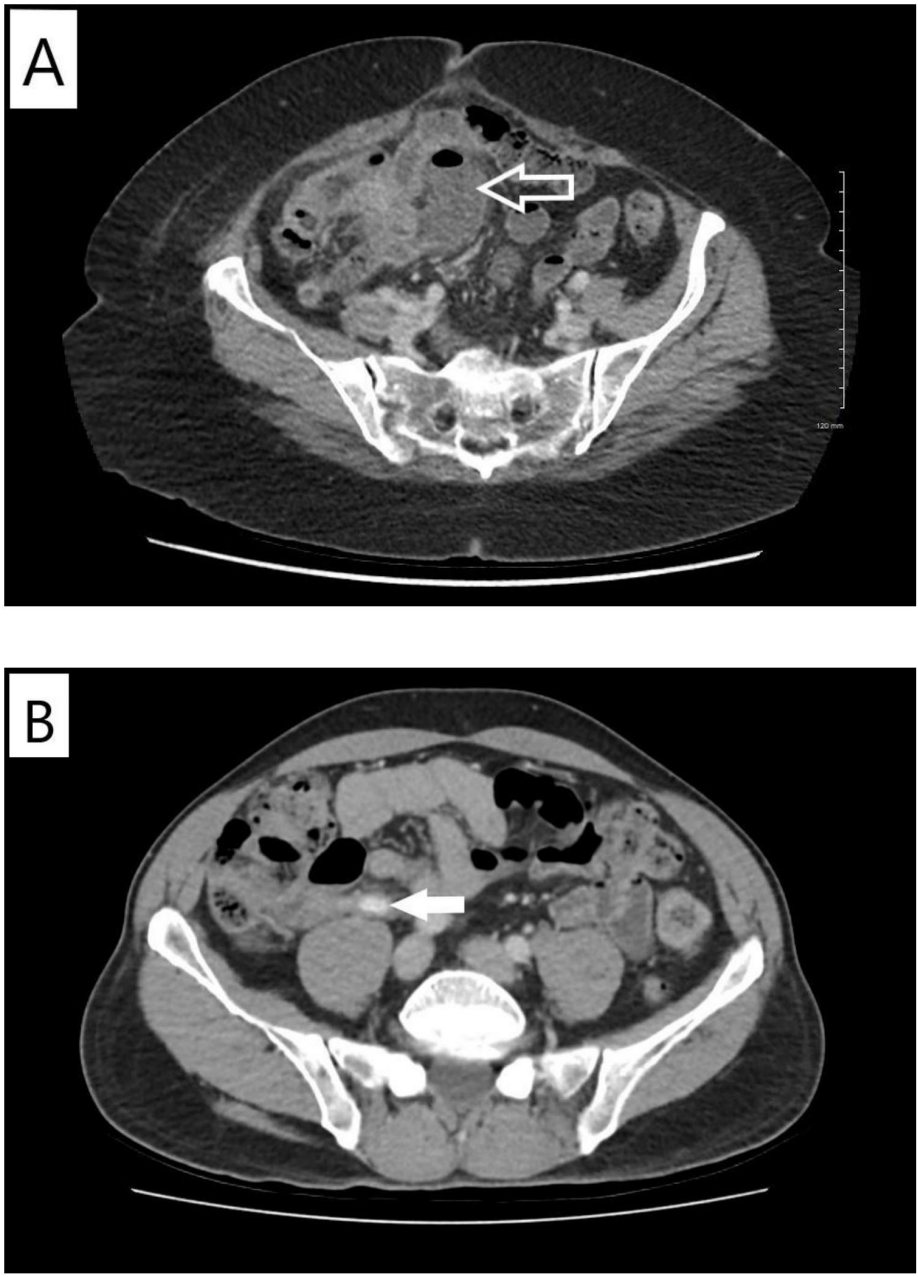
Computed tomographic imaging findings of appendicitis. **(A)** Case X. Contrast-enhanced computed tomography scan of the abdomen and pelvis performed in September 2020. Arrow indicates loculated fluid collection with gas in the expected location of the appendix likely representing contained perforation. **(B)** Case Y. Contrast-enhanced computed tomography scan of the abdomen and pelvis performed in January 2021. Arrow indicates dilated proximal-mid appendix with peri-appendiceal fat stranding.

In September 2020, the patient presented with transient right lower quadrant (RLQ) abdominal pain, nausea, emesis, constipation, and non-specific sweating episodes. Repeat staging scans in the following week showed ongoing radiographic improvement of metastatic disease; however, there was an additional reporting of stranding around the expected location of appendix with apparent loculated fluid collection representing a contained perforation (**Figure 1A**). The appendix itself was not clearly visualized. The patient received oral antibiotics with amoxicillin/clavulanate for 14 days in response. Her symptoms resolved and a repeat CT abdomen a few weeks later showed clearance of the fluid collection with some residual inflammation.

**Figure 2:**
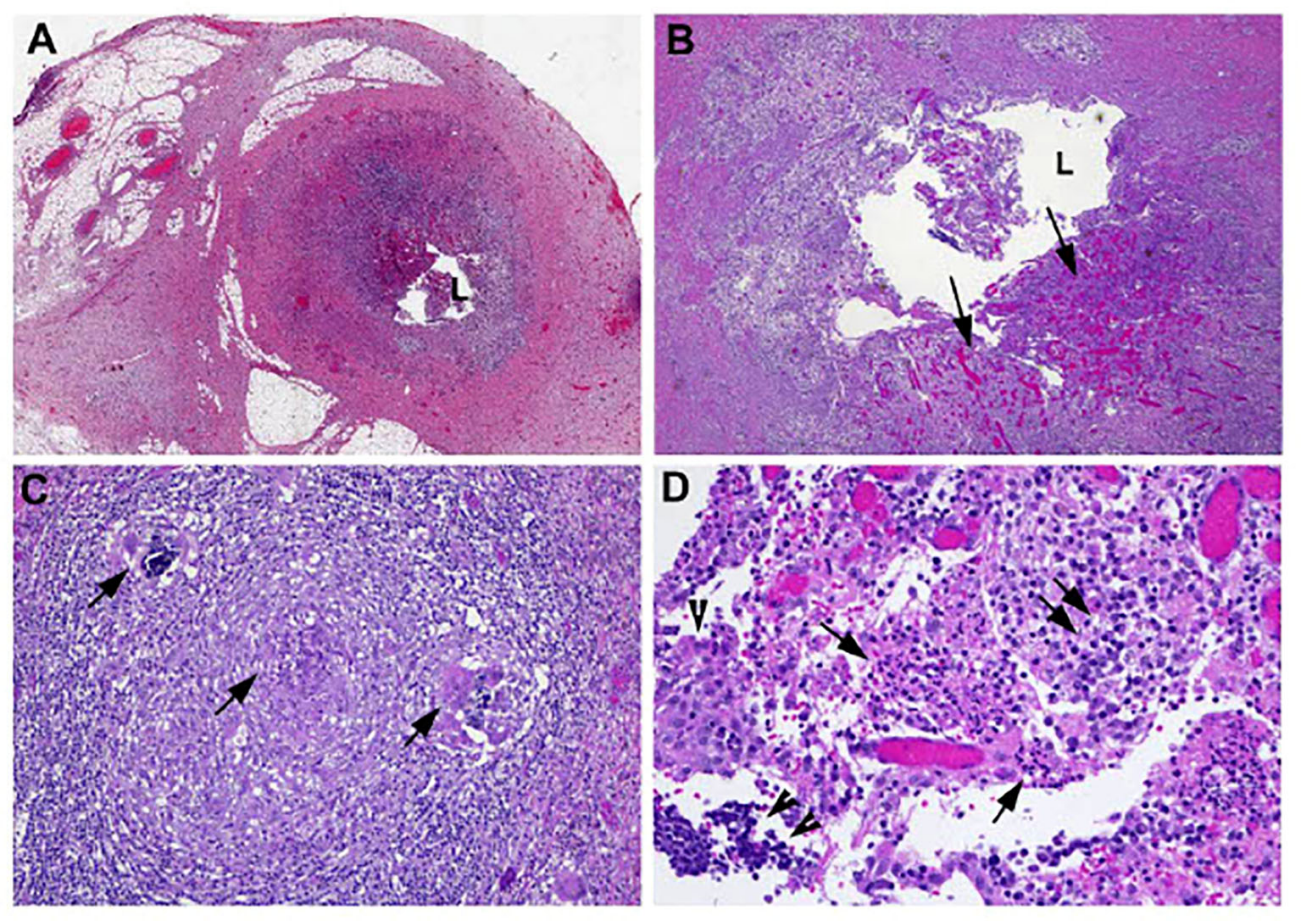
Microscopic examination of the appendectomy specimen showed focal areas of mucosal erosion with neutrophilic infiltrate and submucosal granulation tissue formation. In addition, there was also small granuloma formation in the submucosa. Morphologic features reflect subacute/resolving appendicitis. Photomicrographs show **(A)** cross-section of the appendix with focal erosion of mucosa (magnification 20×); **(B)** with granulation tissue formation (arrows) in submucosa (magnification 100×); **(C)** occasional granulomas in submucosa (arrows, magnification 200×); and **(D)** mixed neutrophils (arrows), plasma cells (double arrows), and lymphocytes (double arrowheads) infiltrating epithelial cells (single arrowhead) (magnification 400×).

**Figure 3:**
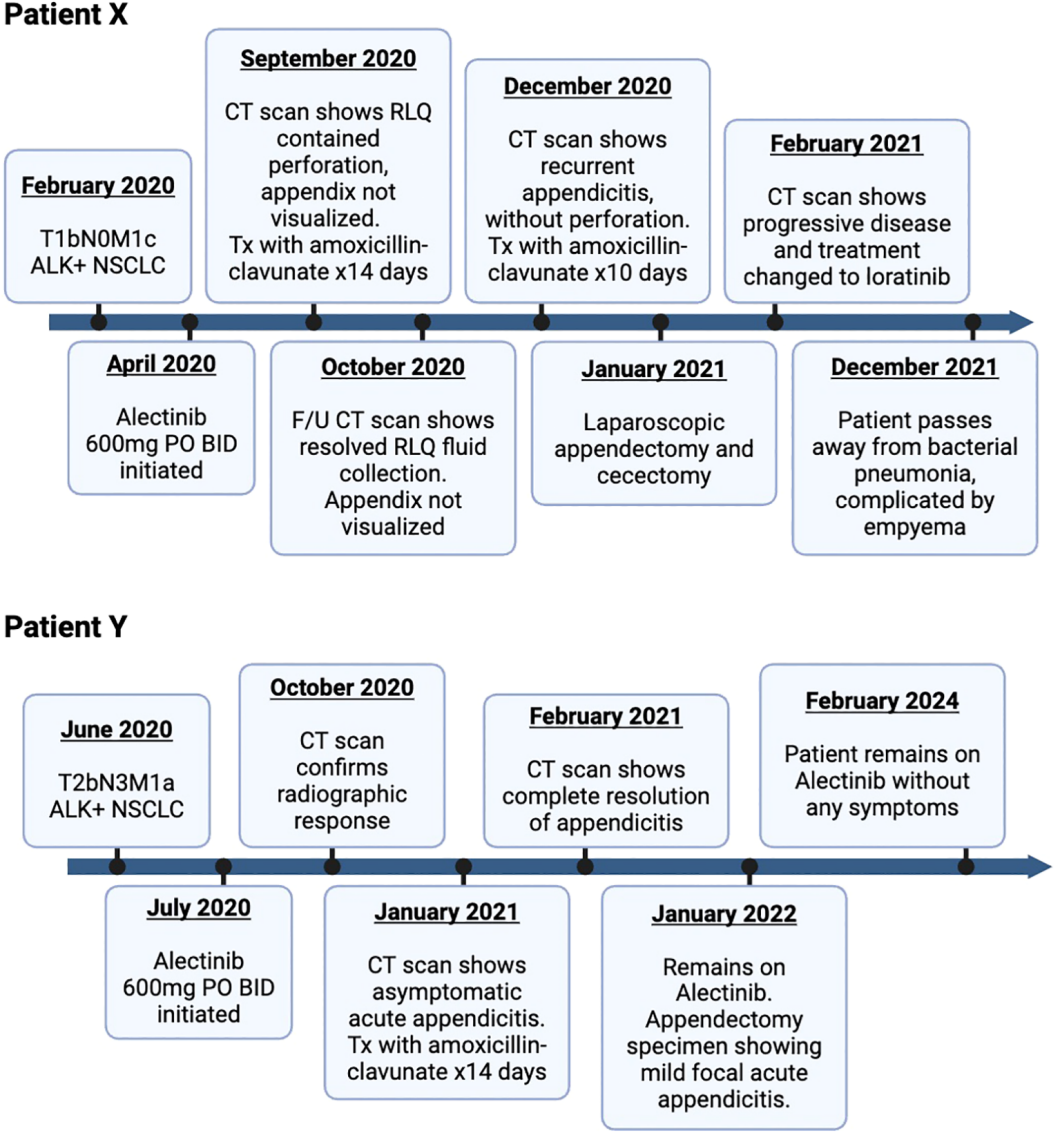
Timeline of patient X and Y: diagnosis and treatment for ALK-positive lung adenocarcinoma with alectinib. ALK, anaplastic lymphoma kinase; NSCLC, non-small cell carcinoma; BID, twice a day; CT, computed tomography; RLQ, right lower quadrant.

Unfortunately, the patient re-presented 6 weeks later with periumbilical abdominal pain radiating to RLQ, resulting in a vagal episode. A repeat CT scan showed peri-appendiceal inflammatory stranding in RLQ consistent with recurrent appendicitis without evidence of perforation or new/worsening metastatic disease. The patient was treated with antibiotics for 10 days, followed by a laparoscopic appendectomy and cecectomy in January 2021. Surgical pathology (**Figure 2**) confirmed findings suggestive of a prior episode of appendicitis, with no evidence of malignancy. There was no interruption to her alectinib regimen, and she continued to receive alectinib until disease progression in February 2021. She was subsequently switched to second-line loralatinib. Her disease subsequently continued to progress until she ultimately passed away in December 2021 following a bacterial pneumonia complicated by empyema. A detailed timeline of case study X is presented in **Figure 3**.

### 2.2 Case study Y

Patient Y is a 58-year-old previously healthy man with no smoking history diagnosed in June 2020 with stage IV metastatic lung adenocarcinoma after presenting with cough and dyspnea. A detailed timeline of case study Y is also presented in **Figure 3**. Initial staging investigations revealed a malignant pleural effusion, but no extra-thoracic metastatic disease. Cytology from pleural fluid confirmed adenocarcinoma with an ALK fusion. Treatment was started with alectinib 600 mg PO BID in July 2020, with immediate symptomatic improvement. Response was confirmed on restaging CT scans in October 2020. There was no clinical or laboratory evidence of major toxicity until a CT scan of the abdomen and pelvis in January 2021 showed evidence of acute appendicitis in the absence of any abdominal pain, nausea, emesis, diarrhea, or fever (**Figure 1B**). He was given antibiotics and referred to general surgery without any interruption or modifications to alectinib. Repeat CT scan the following month showed complete resolution of the appendicitis. He remained asymptomatic with an ongoing response to alectinib and underwent an elective appendectomy on 24 January 2022. Histological examination of the resected specimen diagnosed the patient with mild focal acute appendicitis. Follow-up CT scans in February and May 2022 confirmed stable metastatic disease. At the time of writing, the patient continued to receive alectinib and remains asymptomatic (**Figure 3**).

## 3 Discussion

Metastatic carcinoma is a rare cause of acute appendicitis. Several case reports have described acute appendicitis in patients due to extrinsic compression of appendiceal lumen by metastases, metastatic tumor deposit in the appendix, or as a complication of chemotherapy-induced myelosuppression (8). Specifically, there have been approximately 13 documented cases of lung cancer metastases to the appendix causing appendicitis, including three patients where appendicitis was the presenting complaint (9). From a cancer treatment perspective, patients have been reported to experience appendicitis following administration of immune checkpoint inhibitors, raising suspicion as a possible adverse side effect (10, 11).

Several gastrointestinal complications have been reported as adverse events on ALK inhibitors, such as gastrointestinal perforations on crizotinib, alectinib, and ceritinib (12). However, appendicitis specifically has not been a well-documented side effect of these same inhibitors. In ALK-positive lung cancers, only one study has reported two alectinib-treated patients developing acute appendicitis and appendicitis with perforation (7). Appendicitis has been occasionally reported in patients receiving other ALK inhibitors such as crizotinib and brigatinib (13, 14). Kawata et al. reported three cases with brigatinib-associated appendicitis in the ALTA trial (14). However, no cases of appendicitis have yet been reported in patients treated with lorlatinib or ceritinib. Lastly, no grade 5 adverse events were noted due to appendicitis. In all cases in the current literature, management details and outcome are unknown.

In both our cases, the patients presented with subacute or no symptoms related to appendicitis, with the diagnosis suggested based on imaging findings. Symptomatic acute appendicitis is typically first investigated by ultrasound of the abdomen, then confirmed with CT imaging (15). In our two cases, diagnosis of appendicitis was made during restaging CT scans, both in the presence and in the absence of symptoms. This suggests that, in the absence of a causal link at this time, routine restaging could represent a reasonable screening test for treatment-related appendicitis. Common etiologies of appendicitis were excluded by CT imaging in both our cases including bile duct obstruction by gallstones or another lesion, both of which were absent in our patients. Histopathological examination of the appendectomy specimen in our first patient showed that there was no evidence of metastatic disease as the etiology of appendicitis (**Figure 2**). For treatment, antibiotics with appropriate intra-abdominal microbe coverage and subsequent appendectomy were effective treatment. Both patients were able to continue alectinib treatment uninterrupted.

Altogether, these case reports do not rule out the possibility that appendicitis pathological processes may be driven by alectinib. While the exact mechanism is unclear, alectinib and lorlatinib have been shown to downregulate levels of certain matrix metalloproteinases (MMPs) (16). Given that these MMPs, which typically serve to degrade extracellular matrix and control inflammatory processes (17), are more frequently dysregulated in cases of acute appendicitis (18), it is possible that alectinib may lead to appendicitis through MMP imbalance. As we describe only two cases of this phenomenon, there is currently no clear cause–effect relationship and therefore it is plausible that these events happened only by chance. It would therefore be important for other centers to continue to report any observation of this phenomenon before initiation of a formal investigation into a causal analysis between alectinib and appendicitis. While no immediate changes to clinical practice are warranted with respect to monitoring protocols for appendicitis, we believe that raising awareness about this possible link allows for its timely identification should cases continue to recur in the future.

## 4 Conclusion

Appendicitis is a rare but important differential diagnosis in patients presenting with gastrointestinal symptoms receiving alectinib. Whether these are sporadic or represent a possible class effect is unclear. Timely interventions including intravenous antibiotics and appendectomy allowed for successful treatment without disruption to alectinib therapy.

## Data Availability

The original contributions presented in the study are included in the article/supplementary material. Further inquiries can be directed to the corresponding author.
